# Reply to the commentary on “Cross-sectional associations between dietary intake of polyunsaturated fatty acids, physical function, and sarcopenia in community-dwelling older adults”

**DOI:** 10.1016/j.jnha.2025.100478

**Published:** 2025-01-16

**Authors:** Hélio José Coelho-Júnior, Alejandro Álvarez-Bustos, Anna Picca, Francesco Landi, Emanuele Marzetti

**Affiliations:** a Fondazione Policlinico Universitario Agostino Gemelli, IRCCS, Rome, Italy; b Department of Geriatrics and Orthopedics, Università Cattolica del Sacro Cuore, Rome, Italy; c Biomedical Research Center Network for Frailty and Healthy Ageing (CIBERFES), Institute of Health Carlos III, Madrid, Spain; d Department of Geriatrics, Hospital Universitario de Getafe, Madrid, Spain; a Biomedical Research Center Network for Frailty and Healthy Ageing (CIBERFES), Institute of Health Carlos III, Madrid, Spain; b Instituto de Investigación IdiPaz, Madrid, Spain; a Fondazione Policlinico Universitario Agostino Gemelli, IRCCS, Rome, Italy; b LUM “Giuseppe Degennaro” University, Bari, Italy; a Fondazione Policlinico Universitario Agostino Gemelli, IRCCS, Rome, Italy; b Department of Geriatrics and Orthopedics, Università Cattolica del Sacro Cuore, Rome, Italy

Dear Editor,

We read the commentary wrote by Borazan et al. [1]. The article does not substantially contribute to the discussion of our work, as it merely highlights methodological approaches already recognized as major limitations.

Particularly, authors stressed that the use of dietary recalls, specifically three-day dietary records (3DDR), should be prioritized over other instruments, such as the food frequency questionary (FFQ), used in our study, as they represent more valid and reliable tools to assessing food consumption in older adults. These assumptions are superficial and readily contested.

The notion of “best” instrument (or “more valid and reliable” in scientific language) depends on the context and purpose in which it is utilized. FFQs, for instance, are a subjective method widely used in epidemiological studies [2] and frequently mentioned as valid instruments for this purpose [2], given its easy application, ability to properly assesses usual dietary intake, low-cost, and reduced time consumption [2]. On the other hand, although 3DDR are very useful to clinical practice and allow the estimation of detailed dietary data, they are also a subjective measure, which depends on participants’ motivation and literacy levels [2]. Furthermore, they are considerably time-consuming and difficult to interpret, if significant day-to-day variations in records are observed [2]. As such, 3DDR might not be the more suitable approach for investigations involving single-point data collection, with numerous participants, and assessing several health parameters, as the Lookup 7+ project.

Another point raised by authors, susceptible to disagreement, is the limitations associated with the use of a formula based on calf circumference to estimate appendicular skeletal muscle mass. The use of gold-standard body composition assessment tools (e.g., dual energy X-ray absorptiometry [DEXA] and magnetic resonance imaging [MRI]) is impractical for most investigations, including ours. The use of bioimpedance (BIA) devices could be an alternative to these methods. However, the fact that participants of the Lookup 7+ project were examining while attending events hampers their capacity to be adequately prepared for BIA evaluation.

Notably, contrary to what the authors stated, the European Working Group on Sarcopenia in Older People [3] mentions *ad verbum* that *calf circumference measures may be used as a diagnostic proxy for older adults in settings where no other muscle mass diagnostic methods are available*. Furthermore, the fact that this instrument has potential to discriminate people at higher risk of adverse events, has been validated individually or in the combination with dynapenia (i.e., sarcopenia) against many negative health outcomes, offers similar predictive value, when compared to established sarcopenia operationalization methods, and has proposed normative values [4], indicates its relevance, supports its utilization in scientific and clinical practices, and explain its recommendations by panels of experts [5].

We acknowledge that sarcopenia is recognized as a biological substrate of physical frailty. However, it remains unclear how assessing frailty would have enhanced our results. Indeed, since frailty is an expected consequence of the progression of sarcopenia, it is reasonable to assume that it could be evaluated as a follow-up outcome. In this context, the lack of significant associations between omega-3 dietary intake and sarcopenia suggests that any potential correlations with frailty would likely be minimal.

Understanding the importance, strengths, and limitations of the many assessment tools used in the context of geriatric is essential for comprehensively understanding and critically interpreting evidence. The fact that gold standard or costly and time-intensive assessment tools was not used in an observational study may not warrant substantial criticism. Actually, the continuous research on the development and improvement of easy-to-apply tools that might effectively represent measures of health parameters is mandatory to allow the monitoring of older people around the world, including those from low-income countries. Otherwise, knowledge and care will be limited to small portions of the population. Furthermore, a more comprehensive understanding of the subject under discussion may be necessary to offer meaningful and serious insights.

## Declaration of competing interest

The authors declare that they have no known competing financial interests or personal relationships that could have appeared to influence the work reported in this paper.
